# Exploring relation types for literature-based discovery

**DOI:** 10.1093/jamia/ocv002

**Published:** 2015-05-12

**Authors:** Judita Preiss, Mark Stevenson, Robert Gaizauskas

**Affiliations:** Department of Computer Science, The University of Sheffield 211 Portobello, Sheffield S1 4DP, UK

**Keywords:** literature based discovery, text mining, knowledge discovery, natural language processing

## Abstract

**Objective** Literature-based discovery (LBD) aims to identify “hidden knowledge” in the medical literature by: (1) analyzing documents to identify pairs of explicitly related concepts (terms), then (2) hypothesizing novel relations between pairs of unrelated concepts that are implicitly related via a shared concept to which both are explicitly related. Many LBD approaches use simple techniques to identify semantically weak relations between concepts, for example, document co-occurrence. These generate huge numbers of hypotheses, difficult for humans to assess. More complex techniques rely on linguistic analysis, for example, shallow parsing, to identify semantically stronger relations. Such approaches generate fewer hypotheses, but may miss hidden knowledge. The authors investigate this trade-off in detail, comparing techniques for identifying related concepts to discover which are most suitable for LBD.

**Materials and methods** A generic LBD system that can utilize a range of relation types was developed. Experiments were carried out comparing a number of techniques for identifying relations. Two approaches were used for evaluation: replication of existing discoveries and the “time slicing” approach.^1^

**Results** Previous LBD discoveries could be replicated using relations based either on document co-occurrence or linguistic analysis. Using relations based on linguistic analysis generated many fewer hypotheses, but a significantly greater proportion of them were candidates for hidden knowledge.

**Discussion and Conclusion** The use of linguistic analysis-based relations improves accuracy of LBD without overly damaging coverage. LBD systems often generate huge numbers of hypotheses, which are infeasible to manually review. Improving their accuracy has the potential to make these systems significantly more usable.

## INTRODUCTION

The number of academic papers being published is now so large that researchers are unable to read everything potentially relevant to their research and normally focus only on publications that are directly relevant to their particular specialisation. However, this can lead to novel connections between sub-fields being missed.^2^ Literature-based discovery (LBD) aims to (semi-)automate the process of identifying these connections. A number of possible applications exist, such as: identification of treatments for diseases, drug re-purposing, disease candidate gene discovery, or drug side effect prediction.^3^ For example, Swanson^4^ found a connection between Raynaud's disease and fish oil due to connecting a publication describing the effect of *Raynaud's phenomenon* on *blood viscosity* with a separate publication containing *fish oil*'s effect on the same. This approach to LBD, through an overlap of relationships between terms across multiple publications, is known as the A-B-C model. If the relationship between A and C was not previously known then it is considered an example of “hidden knowledge.” Other techniques have been proposed, for example, discovery patterns which rely on patterns that are matched against documents. The patterns may be either manually created^5^ or inferred from data.^6^ Discovery patterns have proved useful for the discovery of novel drug applications, an application that focuses on a restricted set of concepts and clearly defined relations between them. It is not clear if this technique can be applied to more open ended literature based discovery problems.

LBD systems rely on being able to identify relationships between terms within documents. For example, the A-B-C model relies on the identification of the relationship between A and B, as well as the relationship between B and C. In *closed discovery,* both A, the source term, and C, the target term, are specified, and only the linking terms (with relationships to both A and C) are sought; while *open discovery* explores a much larger space with only the source term being specified and all relationships being pursued. However, identification of relations is a difficult problem and despite the significant amount of research that has been carried out on the topic,^7^^,^^8^ one that has not yet been solved. Consequently, researchers working on LBD have adopted a number of approaches to identifying relations. One simple technique is term co-occurrence, which assumes that terms that are found in the same document are somehow semantically related. This approach is simple to compute but is likely to over-generate relations, as the semantic relation between two terms that do no more than occur in the same document is liable to be very tenuous. An alternative, more complex method is to carry out some sort of linguistic analysis of the text in order to identify related terms. This approach generates fewer relations and the generated relations are likely to signal closer semantic association. However, it requires significantly more computation and the value of the relations identified depends on the accuracy of the linguistic analysis. This paper compares these two approaches to identifying relations within documents and the effect they have within an LBD system. We focus on the A-B-C model due to its generality.

## BACKGROUND AND SIGNIFICANCE

Swanson's discoveries,^4^^,^^9–11^ showed the potential impact of LBD, but also highlighted the scale of the search space.^12^ Within the biomedical domain, knowledge discovery is frequently based on (a subset of) MEDLINE, the US National Library of Medicine’s database of medical journal publications, which in its 2011 release indexed over 19 million articles. To reduce the search space, replication of Swanson’s discoveries has been frequently based on shorter time intervals, such as 1983–85^13^ or 1960–85.^2^

Another approach to search space reduction involves restricting the type of terms that can appear as linking (B) or target (C) terms (in open discovery) in the A-B-C model. A hidden connection to the term *fish oil* is much more informative than a hidden link to the very general term *severe pain*. Term reduction can take the form of removing frequent terms,^14^ restricting target terms by the Unified Medical Language System metathesaurus (UMLS) semantic type,^15–17^ or using association rules.^17^^,^^18^ Medical Subject Heading terms have also been used as underlying concepts.^19^

It is not only the number of terms that determines the complexity of the task. The number of hidden connections will also be proportional to the number of relations between these terms. Most approaches follow Swanson’s work in employing co-occurrence based relations,^20^ but other semantic based approaches are possible.

Two evaluation methods for LBD systems have been described in the literature. *Replication of previous discoveries* measures an LBD system’s ability to reproduce discoveries made by previous LBD systems, normally those described by Swanson.^2^^,^^21^ The *timeslicing approach* evaluates LBD systems by comparing the hypotheses that are generated by analysing the set of documents published before some cut-off-date against the connections that are explicitly stated in the literature published after that date.^1^

## MATERIALS AND METHODS

We implemented an LBD system based on the A-B-C model that can be configured to use different relations between terms and used it to carry out experiments comparing a range of different types of relations. For all our experiments, we use UMLS^22^ Concept Unique Identifiers (CUIs) as terms (as identified by MetaMap), although the system is not limited to these and can work with terms directly (see discussion in Section “Focus on Scale” below).

The LBD system assumes the existence of a binary relation *R* between terms. Let $a_{ij}$ of the term-term matrix *A* describe the frequency with which term $t_{i}$ is related to term $t_{j}$ in the document collection (i.e., the frequency of $t_{i}Rt_{j}$). Any non zero terms in

$$norm\left(A^{2}\right)-norm\left(A\right),$$

where norm converts all non zero values to 1, represent indirectly related concepts which are connected through one interlinking term. The following aspects of the matrix can be varied:

1. **relation**: The relation used to describe the relationship between terms – $t_{i}$ and $t_{j}$ may be related under one relation, but not another.
2. **weight**: The weight, rather than frequency, assigned to each relationship (which will yield a resulting weight for each hidden connection, allowing a ranking to be constructed).
3. **size**: The size of the collection from which the matrix is built—this can be restricted to a particular time interval or possibly even particular category of abstracts.

We explored 6 different types of relations, the first three of which are based on co-occurrence and the remaining three on linguistic analysis. The relations were used to populate the matrix *A* in our LBD system with weights as described below. The collection size was changed in line with evaluation type.

**c-doc:** Co-occurrence of terms based on the entire document (in this case, a document is an abstract). Pairs of terms are considered to co-occur if they are found in the same document and the strength of their co-occurrence is based on the number of documents in which they co-occur. Using this approach the number of times the two terms $a_{i}$ and $a_{j}$ appear within the same documents is stored in the position $a_{ij}$ of *A*.
**c-sent:** A more restrictive approach is to consider terms to co-occur if they are found in the same sentence within a document (abstract). In this case, the strength of co-occurrence between a pair of terms is based on the number of documents which contain at least one sentence in which both terms occur.
**c-title:** The final co-occurrence-based relation uses only the titles of documents. Pairs of terms are considered to co-occur if they are found in the same document title and the co-occurrence strength is based on the number of titles in which they co-occur.
**SemRep:** SemRep,^23^ a publicly available tool, extracts subject-relation-object triples (such as *X* treats *Y*) from biomedical text using underspecified syntactic processing and UMLS domain knowledge. Position $a_{ij}$ stores the count of the triple $a_{i}Ra_{j}$.
**ReVerb:** The publicly available ReVerb Information Extraction system^24^ extracts binary relations expressed by verbs based on imposed syntactic and lexical constraints. Position $a_{ij}$ contains the count of occurrences of the relation $a_{i}Ra_{j}$.
**Stanford:** The publicly available Stanford parser^25^ generates typed grammatical relations, such as subject, between pairs of words extracted from phrase structure trees. A number of grammatical relation patterns were manually constructed and the number of times $a_{i}$ is linked to $a_{j}$ throughout the document collection is stored in $a_{ij}$.

**Figure 1:**
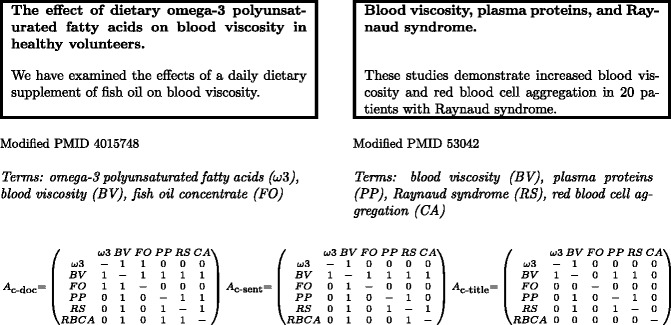
A small scale example illustrating the difference between the co-occurrence based relations.

Figure 1 shows the difference between c-sent, c-doc, and c-title on a small scale example, a document collection consisting of two very short abstracts. While none of the *A* matrix instances contain a link between FO and RS, it can be seen that in this example the relevant $A^{2}$ field will be non-zero for c-doc and c-sent, and the link will be suggested.

## FOCUS ON SCALE

As the quantity of data used for LBD increases, so does the amount of hidden knowledge generated from it. Large quantities of hidden knowledge are difficult to evaluate and may not be helpful to users of the LBD system. Past research addressed this issue using various techniques, including: filtering terms prior to generation, restricting either the time period from which hidden knowledge is generated or the segment of the abstract that knowledge is drawn from (e.g., titles only) and re-ranking of the subsequently produced hidden knowledge. While these approaches make the task more computationally tractable, there is an increased risk of discarding important links or terms, or failing to include crucial knowledge from a previous time period.

### Term reduction

Term reduction has been explored in a number of different forms: Swanson et al.^26^ used a semi-automatically created stoplist of 9500 terms. They also carry out further reduction at an earlier stage: the literature for terms “A” and “C” is pre-filtered by subject heading—for term “X,” the literature only includes abstracts in which “X” is the Medical Subject Heading subject heading and appears in the title. While both techniques decrease complexity and reduce the number of spurious links, restricting the literature on a per term pair basis requires prior knowledge of intended search terms, and therefore needs tuning prior to execution.

A more general filtering approach is suggested by Weeber et al.^2^ who filter out noncontent words by switching LBD from terms to UMLS^22^ CUIs, which only exist for terms appearing in the UMLS Metathesaurus. They use the MetaMap tool^27^ to identify CUIs in documents; a further advantage of MetaMap is its ability to identify multiword units and map these to CUIs—both features of MetaMap greatly reduce the number of ‘terms' given to the LBD system, and help the system avoid spurious connections due to term ambiguity.

Within our large scale, open discovery system, we employ MetaMap as outlined in,^2^ to remove non content words and identify multiwords, and we carry out further term reduction as follows:

1. CUIs which appear in many abstracts are removed. Setting the threshold to 150,000 abstracts results in the removal of 924 terms. This value was manually determined so no obviously “useful” terms were discarded.
2. UMLS contains a list of pairs of CUIs believed to be synonyms, for example, C0034734 (Raynaud disease) and C0034735 (Raynaud Phenomenon). Merging synonymous CUIs allow (a) the retrieval of more hidden knowledge if either is the “A” term, and (b) the potential creation of more hidden knowledge if these terms occur as linking terms (since “A” connected to C0034734 and “C” connected to C0034735 would not have been recognized as being indirectly related previously). This reduces the 561,155 CUIs in UMLS to 540,440 CUI equivalence classes.
3. The UMLS Semantic Network consists of 133 semantic types, a type of subject category, and each CUI is assigned a type. Many of these types are unhelpful for knowledge discovery (e.g., *geographic area* or *language*). Seventy semantic types (which can be viewed at Online Supplementary Appendix A) were manually identified as not being useful, leading to the removal of a further 121,284 CUIs.

## RESULTS AND DISCUSSION

Two evaluations are performed: (1) replication of existing discoveries and (2) timeslicing.

### Replication of existing discoveries

From the LBD literature we identified seven separate discoveries that have previously been used for replication experiments. The time segments from which these were derived are included whenever they could be found in the original paper and used for our experiments:

1. A connection between *Raynaud disease* and *fish oil* was found using Medline articles from three periods: 1983–1985,^21^ 1980–1985,^13^ and 1960–1985.^2^ We present results from the 1960 to 1985 period.
2. A connection between *Somatomedin C* and *arginine* was identified using Medline articles from 1960 to 1989.^28^ (*Somatomedin C* and *arginine* appear together in 27 abstracts which are removed.)
3. A link between *migraine disorders* and *magnesium* was derived from articles in the range 1980–1984.^13^
4. *Magnesium deficiency* was linked to *neurologic disease.*^29^
5. A link between *Alzheimer's* and *indomethacin* based on Medline articles between 1966 and 1996.^30^ (The six abstracts mentioning both were removed.)
6. A link between *Alzheimer's disease* and *estrogen.*^31^ (25 abstracts mentioning both are removed.)
7. A link between *schizophrenia* and *Calcium-Independent Phospholipase A2* based on 1960–1997 Medline.^32^ (One abstract contained both terms.)

Table 1 presents the results of the replication discovery experiments. The table shows the number of linking terms that are identified based on the SemRep, ReVerb, and Stanford-based relations. The LBD system identifies the hidden knowledge in all cases where at least one linking term is identified. The results show that the existing discoveries can be replicated in the majority of cases. The SemRep relations replicate all seven discoveries, and generally identify several linking terms. The other two relations, ReVerb and Stanford, each replicate five of the discoveries. There appear to be fewer linking terms for the discoveries that are not identified by these two relations. These results demonstrate that relations based on linguistic analysis can replicate a range of existing discoveries in the majority of cases.

**Table 1:** Number of linking terms for replication of existing discoveries with synonym merging and semantic type filtering

	SemRep	ReVerb	Stanford
RD – fish oil	4	0	1
Somatomedin C – Arg	130	22	27
Migraine – Mg	47	3	13
Mg deficiency – ND	43	5	0
AD – estrogen	331	64	76
AD – INN	234	47	49
Schizophrenia – Ca2+iPLA2	13	0	0

Results for the co-occurrence-based relations are not included since searching through their output is impractical given the volume of hidden knowledge they generate (see discussion in “Timeslicing” section). However, two of the co-occurrence based relations (c-doc and c-sent) are guaranteed to generate all of the relations that are generated by the approaches presented in the table and will therefore identify all of the existing discoveries.

The amount of hidden knowledge generated from ReVerb is consistently lower than that generated by the other relations. ReVerb relationships center around a verb. However, a substantial amount of information in Medline is contained within the title, which is, in almost all cases, missing a verb, and thus no ReVerb connections arise from it. This is frequently the cause of the low number of linking terms produced by this relation.

Every piece of hidden knowledge is generated by a set of linking terms which connect the “A” and “C” terms. While a connection may be found between the replication source and target terms, examining the linking terms reveals the value of filtering; for example, the terms linking *Raynaud's* and *fish oil* prior to synonym merging and semantic type filtering are found to be CUIs corresponding to *patient* and *volunteer helper* and the frequently cited *blood viscosity* link is missing. Based on these linking terms, the connection should be discarded. However, when synonyms are merged the list of linking terms expands to include *blood viscosity*, *antimicrobial susceptibility, acetylsalicyclic acid, measurin, ecotrin,* and *brain infarction*. Furthermore, restricting by semantic types leads to *patient* and *volunteer helper* being dropped. As an aside, after synonym application and semantic type filtering the amount of hidden knowledge (i.e., number of linked term pairs “A” and “C” not previously known to be connected) generated by the SemRep relation on the 1960–1985 segment drops from 1,784,468,135 to 223,655,269 (i.e., almost a factor of 8).

### Timeslicing

The replication of an existing discovery is focused on one pair of terms – while the hidden connection is known to have been found previously using some LBD system, there is no guarantee that a new LBD system will make the discovery. However, the correlation between a system's inability to replicate one (or seven) given discoveries (which could be due to a simple misidentification of a multiword, or the failure to spot one related pair of terms) and the overall ability to produce useful hidden knowledge is unclear.

A more representative evaluation would involve identifying more hidden knowledge pairs – an evaluation which would allow a meaningful computation of both precision and recall. This is possible with timeslicing: hidden knowledge is generated from all data up to a chosen cut-off-date and is evaluated against the novel ideas presented in publications after the cut-off date (i.e., the assumption is that some of the hidden knowledge will be “discovered” soon after the inference is possible). However, identifying novel ideas, the “new knowledge,” in publications after the cutoff date is not straightforward: for example, extracting all newly co-occurring pairs of CUIs will clearly give a very large and noisy “gold standard” and will favor LBD quantity over quality. The linguistic principled approaches (SemRep, ReVerb, and Stanford) extract real interactions and should therefore produce more accurate gold standards. Clearly, a piece of new knowledge identified by all three approaches is a highly reliable novel discovery. However, insisting on knowledge identified by all three approaches produces a very small gold standard.

Hidden knowledge is generated from the 2000 to 2005 segment, and an evaluation is performed against a gold standard generated from the 2006 to 2010 segment. Based on relation pairs found after a timeslice at the end of 2005 (removing all relation pairs already seen between the start of Medline and the end of 2005) up to the end of 2010 for the SemRep (1,195,925 relation pairs), ReVerb (486,011 relation pairs), and Stanford (384,934 relation pairs) relations, three different new knowledge gold standards are created:

1. intersection of the three sets of relation pairs (4,106 pairs),
2. relation pairs corresponding to the majority (i.e., appearing in at least two relations) (98,747 pairs), and
3. the union of the three sets of relations (1,964,016 pairs).

Note that the techniques are employed purely to create a gold standard: any LBD approach can be evaluated against the gold standard produced, and should another approach to producing a non-noisy gold standard be available, this could easily be substituted.

Results for all 6 relations, including the three based on co-occurrence (c-doc, c-sent, c-title) and the three that use linguistic analysis (SemRep, ReVerb, and Stanford), are displayed in Table 2. A column describing the relation employed is followed by a column containing the number of hidden knowledge pairs produced by each of the relations. The subsequent columns are paired, the first being the number of hidden knowledge pairs identified in the given gold standard, the second the corresponding $F_{1}$ -measure. The $F_{1}$ -measure is a measure of accuracy which combines both precision (the number of pairs within the gold standard correctly identified over the number of pairs in the gold standard, i.e., “correct”/“gold standard”) and recall (the number of pairs within the gold standard correctly identified over the number of pairs generated, i.e., “correct”/“hidden knowledge”)

$$F_{1}=\frac{\text{2×precision×recall}}{\text{precision+recall}},$$

weighing down the precision of systems producing a large number of spurious pairs which are likely to be unsuitable for users (e.g., returning all possible pairs should result in 100% precision): the highest *F*-measure value for each gold standard is shown in bold and represents the combination which generates the highest proportion of “correct” pairs.

**Table 2:** Timeslice evaluation pre-slice 2000–2005, new knowledge generated from 2006 to 2010, merging synonyms, filtering semantic types.

	Hidden knowledge	Union		Majority		Intersection	
		Correct	*F*	Correct	*F*	Correct	*F*
c-doc	14 601 340 987	762 474	1.04e-04	25 089	3.44e-06	954	1.31e-07
c-sent	5 697 603 946	1 104 869	3.88e-04	41 147	1.44e-05	1485	5.41e-07
c-title	786 977 001	1 392 441	3.53e-03	68 393	1.74e-04	2808	7.14e-06
SemRep	197 590 213	1 268 934	1.27e-02	74 508	7.54e-04	3781	3.83e-05
ReVerb	91 950 221	1 068 498	2.28e-02	66 070	**2.39e-03**	3314	7.21e-05
Stanford	74 442 449	885 203	**2.32e-02**	60 120	1.61e-03	3049	**8.19e-05**

While the co-occurrence approaches clearly return a larger proportion of the gold standard, this is at the expense of generating a much larger volume of hidden knowledge over all. (Note that the lower number of gold standard pairs returned by c-doc vs c-title is genuine: the term-term *A* matrix representing the frequency of occurrence of each related pair, see “Materials and Methods,” section will be much less sparse for c-doc than c-title due to the volume of data included. This results in a more populated $A^{2}$ for c-doc than c-title, but removing the large number of previously related pairs (norm (*A*)) dramatically reduces the number of non zero pairs in norm($A^{2}$) – norm(A).) The *F*-measure shows the complete picture: the semantic knowledge based relations outperform the co-occurrence information each time, and the best such relation is at least 10 times better than the best co-occurrence relation.

The importance of reducing the amount of spurious hidden knowledge candidates cannot be underestimated. Table 3 depicts the number of hidden knowledge pairs generated using each of the 6 relations (# pairs column) as well as the average (mean, median, and mode) number of hidden knowledge candidates per term (the “Terms” column depicts the number of distinct terms appearing in any relation instance—co-occurrence includes most of the terms present in Medline as all pairs are related, while less productive relations involve fewer terms). These figures indicate the average amount of hidden knowledge a user will need to evaluate when they use an LBD system for hypothesis generation. Table 3 also shows that these figures are 1 to 2 orders of magnitude higher when the co-occurrence based relations are used. Note that without synonym merging and semantic type filtering, the amount of hidden knowledge is even larger: c-title yields a total of 9 921 824 584 pairs with a mean of 20 435 pairs per term and c-doc produces 86 955 899 148 with a mean of 179 091 pairs.

**Table 3:** Hidden connection breakdown (with synonym merging and semantic type filtering).

	No. of pairs	Terms	Mean	Median	Mode
	2000–2005				
c-doc	29 202 681 794	233 446	60 145	117 127	78 405
c-sent	11 395 207 892	227 869	50 007	35 071	10 987
c-title	1 573 954 002	138 622	11 354	5679	3
SemRep	395 180 426	88 525	4464	1734	1
ReVerb	183 900 442	90 742	2027	662	1
Stanford	148 884 898	71 389	2086	685	1

## CONCLUSION

LBD systems rely on the identification of relations between terms mentioned within documents. In the previous literature on LBD, various approaches have been explored that vary in terms of the nature of the relations between terms that they identify, in particular whether they simply determine term–term co-occurrence within the same document or same sentence, or whether they perform linguistic analysis. This paper investigated a range of these approaches to relation identification and studied them within an LBD system.

We found that approaches that use relations extracted through automatic linguistic analysis identify several orders of magnitude fewer instances of hidden knowledge than approaches that use term co-occurrence relations, but that these relations are sufficient to replicate existing discoveries in the majority of cases. In addition, we found that the amount of hidden knowledge generated when the linguistic analysis approaches are used appears to be tractable, that is, an interested user could potentially review it all. This contrasts with the term co-occurrence based approaches where the sheer volume of hidden knowledge produced exceeds human capacity to review it. We conclude that using automated linguistic analysis in relation identification for LBD provides significant benefits, in terms of reducing the number of spurious links identified, while still identifying sufficient links to enable potentially interesting discoveries.

## FUNDING

This work was supported by the Engineering and Physical Sciences Research Council grant number EP/J008427/1.

## COMPETING INTERESTS

None.

## CONTRIBUTORS

All authors designed and conceived of the study. Judita Preiss implemented the system and carried out all experiments. All authors read and approved the final manuscript.

## Supplementary Material

Supplementary Data
