# Supplementary material for: Exploring relation types for literature-based discovery
Source: J Am Med Inform Assoc. 2015 May 12;22(5):987–92. doi: 10.1093/jamia/ocv002 (PMC4986660; doi:10.1093/jamia/ocv002)
Supplement: Supplementary Data [file ocv002_supplementary_data.zip › appendix.docx]

List of removed semantic types:

Organism

Plant

Animal

Vertebrate

Bird

Fish

Reptile

Brown Rat

Human

Anatomical Structure

Embryonic Structure

Fully Formed Anatomical Structure

Tissue

Cell

Cell Component

Body Location or Region

Organism Attribute

Injury or Poisoning

Experimental Model of Disease

Event

Activity

Behavior

Social Behavior

Individual Behavior

Daily or Recreational Activity

Occupational Activity

Health Care Activity

Laboratory Procedure

Diagnostic Procedure

Therapeutic or Preventive Procedure

Research Activity

Molecular Biology Research Technique

Governmental or Regulatory Activity

Educational Activity

Machine Activity

Environmental Effect of Humans

Natural Phenomenon or Process

Entity

Physical Object

Manufactured Object

Medical Device

Research Device

Conceptual Entity

Idea or Concept

Temporal Concept

Qualitative Concept

Quantitative Concept

Spatial Concept

Geographic Area

Molecular Sequence

Regulation or Law

Occupation or Discipline

Biomedical Occupation or Discipline

Organization

Health Care Related Organization

Professional Society

Self-help or Relief Organization

Group

Professional or Occupational Group

Population Group

Family Group

Age Group

Patient or Disabled Group

Group Attribute

Chemical Viewed Functionally

Substance

Functional Concept

Intellectual Product

Language

Sign or Symptom
